# Secretory NPC2 Protein-Mediated Free Cholesterol Levels Were Correlated with the Sorafenib Response in Hepatocellular Carcinoma

**DOI:** 10.3390/ijms22168567

**Published:** 2021-08-09

**Authors:** Fat-Moon Suk, Yuan-Hsi Wang, Wan-Chun Chiu, Chiao-Fan Liu, Chien-Ying Wu, Tzu-Lang Chen, Yi-Jen Liao

**Affiliations:** 1 Division of Gastroenterology, Department of Internal Medicine, Wan Fang Hospital, Taipei Medical University, Taipei 116, Taiwan; 95351@w.tmu.edu.tw; 2 Department of Internal Medicine, School of Medicine, College of Medicine, Taipei Medical University, Taipei 110, Taiwan; 3 School of Medical Laboratory Science and Biotechnology, College of Medical Science and Technology, Taipei Medical University, Taipei 110, Taiwan; m609105001@tmu.edu.tw (Y.-H.W.); b117105021@tmu.edu.tw (C.-F.L.); b614107065@tmu.edu.tw (C.-Y.W.); 4 School of Nutrition and Health Sciences, Taipei Medical University, Taipei 110, Taiwan; wanchun@tmu.edu.tw; 5 Research Center of Geriatric Nutrition, College of Nutrition, Taipei Medical University, Taipei 110, Taiwan; 6 Department of Medical Education, Far Eastern Memorial Hospital, New Taipei City 220, Taiwan; tlc787878@gmail.com

**Keywords:** Niemann-Pick type C2 (NPC2), free cholesterol, sorafenib resistance, hepatocellular carcinoma

## Abstract

Hepatocellular carcinoma (HCC) is the most common primary malignant tumor in the world. Sorafenib is the first-line drug for patients with advanced HCC. However, long-term treatment with sorafenib often results in reduced sensitivity of tumor cells to the drug, leading to acquired resistance. Identifying biomarkers which can predict the response to sorafenib treatment may represent a clinical challenge in the personalized treatment era. Niemann-Pick type C2 (NPC2), a secretory glycoprotein, plays an important role in regulating intracellular free cholesterol homeostasis. In HCC patients, downregulation of hepatic NPC2 is correlated with poor clinical pathological features through regulating mitogen-activated protein kinase (MAPK)/extracellular signal-regulated kinase (ERK) activation. This study aimed to investigate the roles of secretory NPC2-mediated free cholesterol levels as biomarkers when undergoing sorafenib treatment and evaluate its impact on acquired sorafenib resistance in HCC cells. Herein, we showed that NPC2 downregulation and free cholesterol accumulation weakened sorafenib’s efficacy through enhancing MAPK/AKT signaling in HCC cells. Meanwhile, NPC2 overexpression slightly enhanced the sorafenib-induced cytotoxic effect. Compared to normal diet feeding, mice fed a high-cholesterol diet had much higher tumor growth rates, whereas treatment with the free cholesterol-lowering agent, hydroxypropyl-β-cyclodextrin, enhanced sorafenib’s tumor-inhibiting ability. In addition, sorafenib treatment induced higher NPC2 secretion, which was mediated by inhibition of the Ras/Raf/MAPK kinase (MEK)/ERK signaling pathway in HCC cells. In both acquired sorafenib-resistant cell and xenograft models, NPC2 and free cholesterol secretion were increased in culture supernatant and serum samples. In conclusion, NPC2-mediated free cholesterol secretion may represent a candidate biomarker for the likelihood of HCC cells developing resistance to sorafenib.

## 1. Introduction

Liver cancer was the fourth leading cause of cancer deaths worldwide in 2018. It was estimated that about 841,000 new cases are diagnosed and 782,000 deaths occur annually [1]. Primary liver cancer includes hepatocellular carcinoma (HCC), intrahepatic cholangiocarcinoma, and other rare types. In particular, HCC is the most common primary liver malignancy, comprising 75%~85% of all cases [2]. Chronic liver disease and cirrhosis remain the main risk factors for developing HCC [3]. Major risk factors include chronic infection with hepatitis B or C virus, heavy alcohol consumption, obesity, and type 2 diabetes [3]. Sorafenib is used to treat advanced HCC in numerous countries worldwide [4]. Sorafenib is an orally administered multikinase inhibitor that is able to increase the median survival by 3 months compared to a placebo group [5]. Sorafenib is able to inhibit tumor cell proliferation and angiogenesis by blocking the Ras/Raf/mitogen-activated protein kinase (MAPK) pathway, vascular endothelial growth factor (VEGF) signaling pathway, and platelet-derived growth factor receptor signaling [6]. However, it was reported that only about 30% of patients are responsive to sorafenib treatment [7], and about 70% of patients develop acquired resistance within 6 months [8]. Since sorafenib treatment only prolongs survival for a few months and sorafenib resistance develops in most patients, identifying novel biomarkers that can reflect the sorafenib response is urgently needed.

Previous studies found that disturbed cholesterol biosynthesis is considered a hallmark of cancer, and it is essential for the development and progression of a wide variety of cancers [9,10]. The liver is the main organ for maintaining cholesterol homeostasis by de novo synthesis, uptake, storage, and secretion to the blood circulation [11]. However, the role of cholesterol in the progression of HCC remains controversial. A previous study showed that dietary cholesterol caused non-alcoholic steatohepatitis (NASH) and accelerated HCC development [12]. Kim et al. found that increased mitochondrial cholesterol levels were expressed by HCC patients, thereby contributing to chemotherapeutic resistance [13]. Conversely, it was reported that high serum cholesterol levels increased antitumor functions of natural killer cells and reduced the incidence and progression of HCC in mice [14]. Yang et al. demonstrated that cholesterol significantly suppressed the migration and invasion of HCC cells [15]. Meanwhile, suppression of cholesterol biosynthesis by two different components of Chinese herbal medicines was reported to improve the anticancer effect of sorafenib in HCC cells [16,17]. Although the role of cholesterol in affecting HCC progression has been investigated for many years, how cholesterol influences sorafenib sensitivity or the acquisition of sorafenib resistance in HCC remains unclear. The Niemann-Pick type C2 (NPC2) protein is a small soluble glycoprotein that contains a 19-amino acid signal peptide, and it mainly resides within endosome and lysosome compartments [18,19]. Intracellularly, NPC2 binds free cholesterol and regulates intracellular free cholesterol trafficking and homeostasis [19]. An NPC2 deficiency results in the accumulation of free cholesterol in cells [20]. In our previous studies, we found that NPC2 expression was downregulated in fibrotic liver tissues, thereby enhancing hepatic stellate cell activation and proliferation [21,22]. In liver cancer tissues, NPC2 expression was downregulated in HCC patients, and lower NPC2 expression was correlated with higher vascular invasion and later stages of pathological grades [23]. In addition, we also reported that NPC2 regulates HCC cell proliferation, migration, and tumorigenesis by regulating extracellular signal-regulated kinase 1/2 (ERK1/2) activation [23]. However, it is still unclear whether NPC2-mediated free cholesterol homeostasis affects the sensitivity of HCC toward sorafenib treatment or the development of acquired sorafenib resistance. In this study, we investigated the roles of secretory NPC2-mediated free cholesterol levels as biomarkers in patients undergoing sorafenib treatment and evaluated its impacts on acquired sorafenib resistance in HCC.

## 2. Results

### 2.1. NPC2 Downregulation Facilitates Free Cholesterol Accumulation which Weakened Sorafenib Efficacy through Enhancing MAPK/AKT Signaling in HCC Cells

**Figure 1:**
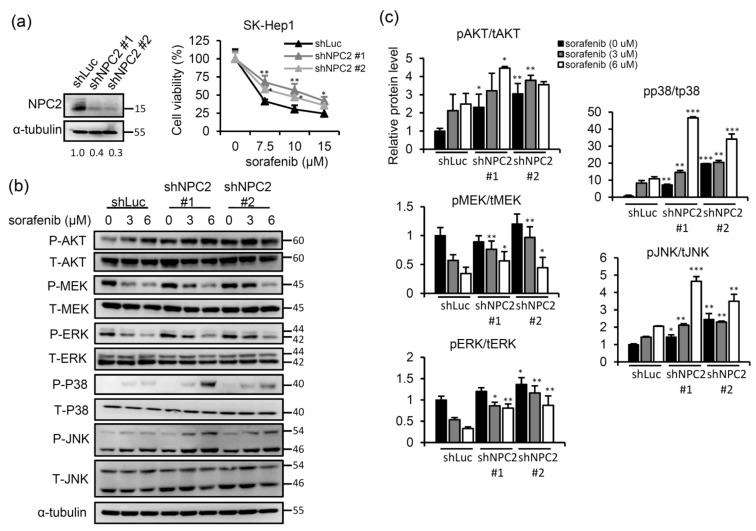
Niemann-Pick type C2 (NPC2) downregulation attenuates sorafenib-induced cytotoxicity through enhancing mitogen-activated protein kinase (MAPK)/AKT signaling in liver cancer cells. (**a**) Western blot analysis was used to confirm the knockdown effect of NPC2. The relative NPC2 intensities are shown under western blot images after normalization with shLuc control intensities. SK-Hep1 cells were treated with 0, 7.5, 10, and 15 μM of sorafenib for 48 h, and cell viability was assessed with an MTT assay. (**b**) SK-Hep1 cells were treated with 0, 3, and 6 μM of sorafenib for 48 h, and protein expression levels of AKT, MAPK kinase (MEK), extracellular signal-regulated kinase (ERK), p38, and c-Jun N-terminal kinase (JNK) were detected by western blotting. α-Tubulin was used as an internal control. These experiments were repeated three times with similar results. (**c**) Western blot images were quantified using Image J software. * *p* < 0.05, ** *p* < 0.01, *** *p* < 0.001 compared to shLuc cells.

In our previous studies, we showed that NPC2 downregulation is related to the development of nonalcoholic fatty liver disease, liver fibrosis, and hepatocellular carcinoma [21,23,24] Nevertheless, the effects of NPC2 expression and intracellular free cholesterol homeostasis on sorafenib sensitivity have not been explored in detail. To study whether NPC2 downregulation influences sorafenib cytotoxicity, a lentivirus carrying small hairpin (sh)RNA targeting NPC2 was used to infect SK-Hep1 cells (Figure 1a). Compared to the shLuc control, NPC2-knockdown (KD) attenuated the cytotoxic effects of sorafenib in SK-Hep1 cells (Figure 1a). Since sorafenib is a multiple kinase inhibitor that targets Raf/MEK/ERK signaling kinases [25], we identified possible mechanisms that contribute to changes in NPC2 downregulation mediation of sorafenib’s cytotoxic effect. Treatment with sorafenib decreased p-MEK and p-ERK expressions in shLuc control cells; however, an NPC2 deficiency in cells enhanced the phosphorylation of MEK and ERK (Figure 1b,c). On the other hand, treatment with sorafenib elevated p-AKT, p-JNK, and p-p38 expressions in shLuc control cells, and these effects were more significant in NPC2-KD cells (Figure 1b,c).

**Figure 2:**
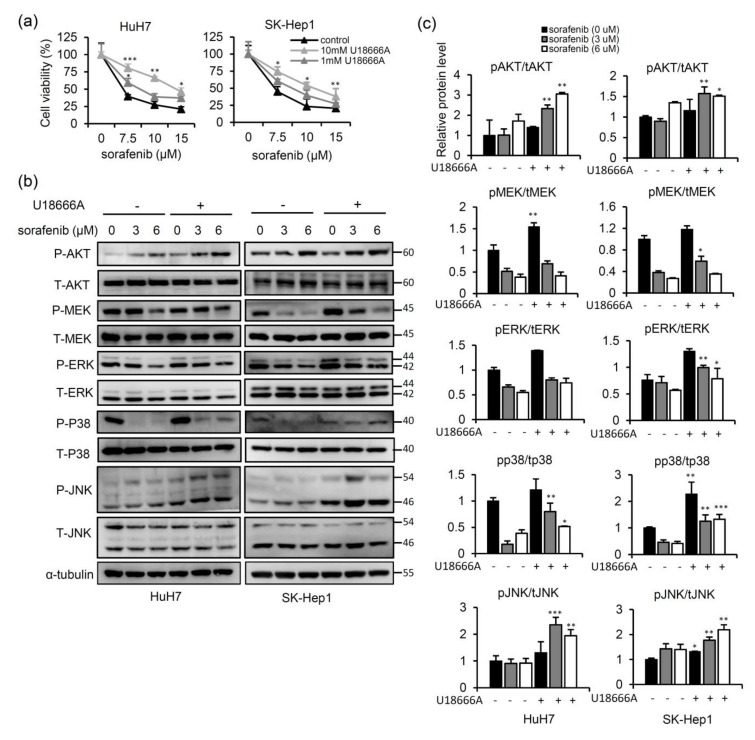
U18666A treatment induces free cholesterol accumulation, which weakened sorafenib-induced cytotoxicity through enhancing mitogen-activated protein kinase (MAPK)/AKT signaling in liver cancer cells. (**a**) HuH7 and SK-Hep1 cells were treated with sorafenib (0, 7.5, 10, and 15 μM) and U18666A (0, 1, and 10 mM) for 48 h and then harvested for an MTT assay. (**b**) HuH7 and SK-Hep1 cells were treated with sorafenib (0, 3, and 6 μM) and U18666A (0 and 10 mM) for 48 h, and protein expression levels of AKT, MAPK kinase (MEK), extracellular signal-regulated kinase (ERK), p38, and c-Jun N-terminal kinase (JNK) were detected by western blotting. α-tubulin was used as an internal control. These experiments were repeated three times with similar results. (**c**) Western blot images were quantified with the Image J software. * *p* < 0.05, ** *p* < 0.01, *** *p* < 0.001 compared to U18666A untreated cells.

Given that abnormal accumulation of intracellular free cholesterol is found in NPC2-deficient cells [19], we next explored whether free cholesterol accumulation in HCC cells affects sensitivity to sorafenib. As shown in Figure 2a, sorafenib treatment led to decreasing cell viability in a dose-dependent manner. However, the combination with different concentrations of U18666A (a free cholesterol transport inhibitor) [26] strongly decreased sensitivity to sorafenib in a dose-dependent manner (Figure 2a). To further clarify the molecular mechanisms that account for the effects of sorafenib treatment on free cholesterol accumulation in HCC cells, we analyzed changes in MAPK/AKT signaling pathways. As shown in Figure 2b, treatment with sorafenib decreased p-MEK, p-p38, and p-ERK expressions, whereas combined U18666A and sorafenib treatment promoted the phosphorylation of MEK, p38, and ERK (Figure 2b,c). In addition, significant activation of p-AKT and p-JNK was found in U18666A-treated cells (Figure 2b,c). These data demonstrated that NPC2 downregulation and free cholesterol accumulation attenuated sorafenib-induced cytotoxicity through enhancing the MAPK/AKT pathways.

### 2.2. NPC2 Overexpression Inhibits MAPK/ERK Signaling and Slightly Enhances Sorafenib-Induced Cytotoxicity

**Figure 3:**
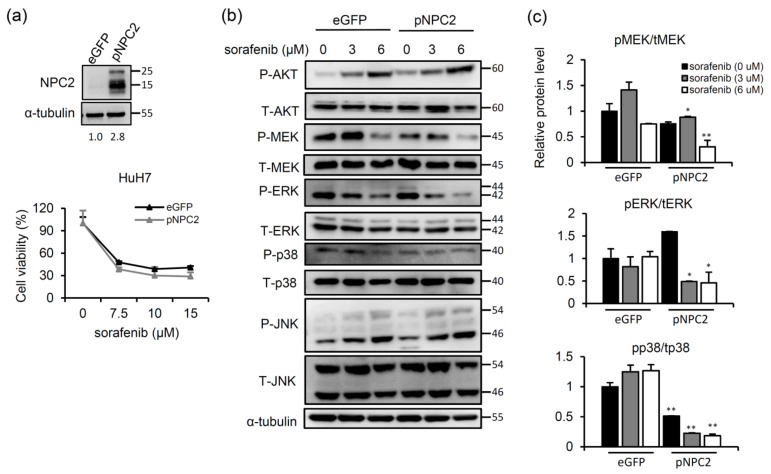
Niemann-Pick type C2 (NPC2) overexpression inhibits mitogen-activated protein kinase (MAPK)/extracellular signal-regulated kinase (ERK) signaling and slightly affects sorafenib-induced cytotoxicity. (**a**) A western blot analysis was used to confirm the effect of NPC2 overexpression. HuH7 cells were treated with sorafenib (0, 7.5, 10, and 15 μM) for 48 h and then harvested for an MTT assay. (**b**) HuH7 cells were treated with 0, 3, and 6 μM of sorafenib for 48 h, and protein expression levels of AKT, MAPK kinase (MEK), ERK, p38, and c-Jun N-terminal kinase (JNK) were detected by western blotting. α-tubulin was used as an internal control. These experiments were repeated three times with similar results. (**c**) Western blot images were quantified with Image J software. * *p* < 0.05, ** *p* < 0.01 compared to enhanced green fluorescent protein (eGFP) cells.

We next examined whether NPC2 overexpression improved sorafenib-induced cytotoxicity. HuH7 cells were infected with a lentivirus carrying the *NPC2* gene to overexpress NPC2 (Figure 3a), and then cells were treated with different concentrations of sorafenib. Cell viability of sorafenib-treated enhanced green fluorescent protein (eGFP)-controlled cells decreased in a dose-dependent manner, whereas NPC2 overexpression slightly enhanced sorafenib-induced cytotoxicity (Figure 3a). Nonetheless, significant inhibition of p-MEK, p-p38, and p-ERK was observed in sorafenib-treated NPC2-overexpressing cells (Figure 3b,c). Meanwhile, p-AKT and p-JNK expressions did not differ between sorafenib-treated eGFP and NPC2-overexpressing cells (Figure 3b). These data imply that although NPC2 overexpression only slightly enhanced sorafenib-induced cytotoxicity, inhibition of MAPK/ERK signaling was observed in NPC2-overexpressing cells.

### 2.3. Treatment with a Free Cholesterol-Lowering Agent Improves Sorafenib’s Ability to Inhibit Tumors in High-Cholesterol Diet-Fed Groups

**Figure 4:**
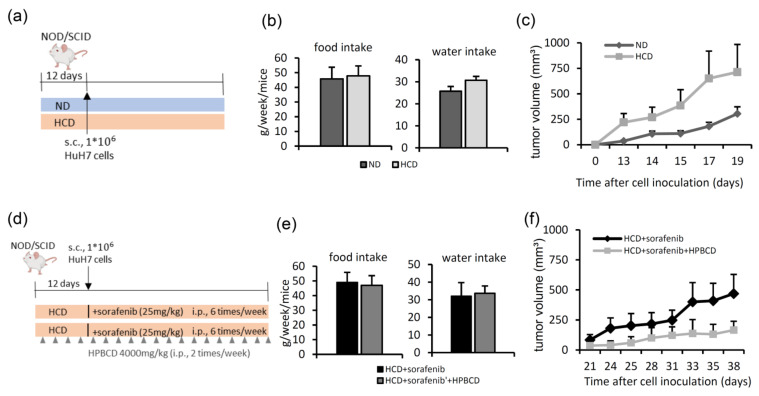
Free cholesterol-lowering agent treatment improves sorafenib’s tumor-inhibiting ability in the group fed a high-cholesterol diet. (**a**) Scheme of the experimental strategy of the effects of a normal diet (ND) and a high-cholesterol diet (HCD) on tumor growth. (**b**,**c**) Average food intake, water intake, and tumor sizes of the mice. All values are expressed as the mean ± SEM (*n* = 5). (**d**) Scheme of the experimental strategy of the effects of free cholesterol-lowering drug (2-hydroxypropyl-β-cyclodextrin (HPBCD)) plus sorafenib in a hepatocellular carcinoma (HCC) xenograft model fed an HCD. (**e**,**f**) Average food intake, water intake, and tumor sizes of mice. All values are expressed as the mean ± SEM (*n* = 5).

To explore the effects of a high-cholesterol diet on the tumor growth rate, NOD/SCID mice were inoculated with HuH7 cells after 12 days of pre-feeding a normal diet or a high-cholesterol diet (Figure 4a). During the diet-feeding period, water intake and food intake did not significantly differ among the mice (Figure 4b). Compared to the group fed a normal diet, the tumor growth rate was higher in the high-cholesterol diet group (Figure 4c). To further evaluate whether treatment with a free cholesterol-lowering agent can improve sorafenib’s efficacy in mice fed a high-cholesterol diet, NOD/SCID mice were inoculated with HuH7 cells after 12 days of high-cholesterol diet feeding, after which the mice were treated with sorafenib the next day. Another group additionally received HPBCD to reduce free cholesterol accumulation (Figure 4d). HPBCD is currently in phase 2 clinical trials investigating its effectiveness in treating NPC2 deficiency-associated free cholesterol accumulation in NPC disease [27]. Throughout the treatment duration, the water intake and food intake did not differ among these mice (Figure 4e). As shown in Figure 4f, HPBCD and sorafenib co-treatment produced significant inhibition of the tumor growth rate compared to that of sorafenib treatment alone. These data demonstrated that a high-cholesterol diet promoted HCC tumor growth, while HPBCD treatment enhanced the tumor-inhibiting ability of sorafenib under high-cholesterol diet feeding.

### 2.4. Sorafenib-Inhibited Raf Signaling Pathway Promotes Secretion of NPC2 and Free Cholesterol in Cell Culture Supernatant

**Figure 5:**
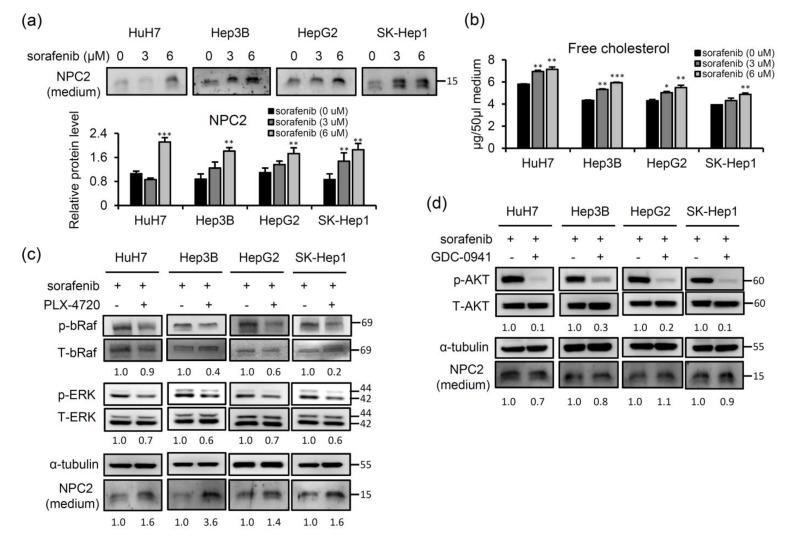
Sorafenib inhibition of the Raf signaling pathway increases the secretion of Niemann-Pick type C2 (NPC2) and free cholesterol into the cell culture supernatant. (**a**,**b**) NPC2 and free cholesterol levels in the cell culture supernatant of hepatocellular carcinoma (HCC) cells treated with the 0, 3, and 6 μM sorafenib for 48 h. * *p* < 0.05, ** *p* < 0.01, *** *p* < 0.001 compared to black bars. (**c**,**d**) HCC cells were treated with 5 μM of sorafenib and 1 μM of a bRaf inhibitor (PLX-4720) or a phosphatidylinositol 3-kinase (PI3K) inhibitor (GDC-0941) for 24 h. Protein expressions of total (T)/phosphorylated (p)-bRaf, T/p-ERK, and T/p-AKT in cells and NPC2 in cultured media were analyzed by western blotting. (**c**) T-bRaf, T-ERK, and α-tubulin was used as a loading control. p-bRaf, p-ERK band, and NPC2 intensities of PLX-4720 (−)/(+) pairs are shown under western blot images after normalization with control intensities. These experiments were repeated three times with similar results.

Since NPC2 plays an important role in regulating intracellular free cholesterol homeostasis via direct binding with free cholesterol and trafficking in intracellular compartments [19] and can also secrete from liver cells [28], we next explored whether the secretion of NPC2 and free cholesterol can be modulated by sorafenib treatment. After 48 h of sorafenib treatment, the levels of the NPC2 protein and free cholesterol in the supernatant had increased in four different HCC cell lines (Figure 5a,b). Since the Ras/Raf/MEK/ERK signaling pathway is the main target by which sorafenib inhibits the proliferation of HCC cells [29], we next evaluated whether NPC2 secretion is responsible for inhibition of this pathway. We used the bRaf inhibitor, PLX-4720, to treat four different HCC cell lines. The results showed that in combination with PLX-4720 treatment, the expression level of NPC2 in cultured medium significantly increased in sorafenib-treated HCC cells (Figure 5c). On the other hand, activation of phosphatidylinositol 3-kinase (PI3K)/AKT is also involved in most cancer-related proliferation signaling [30] and contributes to the development of acquired resistance to sorafenib in HCC [29]. Therefore, we used the PI3K/AKT inhibitor, GDC-0941, to treat four different HCC cell lines. The results showed that while the p-AKT downstream target was inhibited following GDC-0941 treatment, there were no significant changes in NPC2 expression in culture media from four different HCC cell lines (Figure 5d). These results demonstrated that sorafenib treatment induced higher NPC2 secretion which was mediated by inhibition of the Ras/Raf/MEK/ERK signaling pathway in HCC cells.

### 2.5. NPC2 and Free Cholesterol Levels Increase in the Culture Supernatant of Cells with Sorafenib Treatment-Induced Acquisition of Sorafenib Resistance

**Figure 6:**
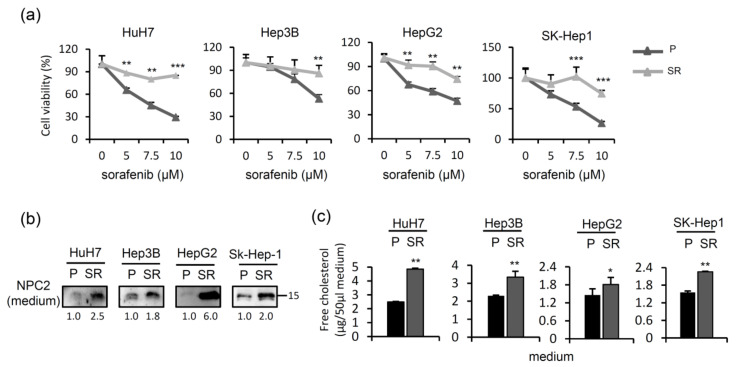
Niemann-Pick type C2 (NPC2) and free cholesterol levels increase in culture supernatant of sorafenib-resistant cells. (**a**) Parental and established sorafenib-resistant cells were treated with indicated doses of sorafenib for 48 h, and cell viability was assessed with an MTT assay. (**b**,**c**) NPC2 and free cholesterol levels in cell culture supernatants of parental and sorafenib-resistant cells. * *p* < 0.05, ** *p* < 0.01, *** *p* < 0.001 compared to parental cells. These experiments were repeated two times with similar results.

Since higher NPC2 and free cholesterol levels are modulated by short-term treatment with sorafenib (Figure 5a,b), we next compared the NPC2-mediated free cholesterol secretion between parental and sorafenib-resistant cells. Hence, we established four sorafenib-resistant HCC cell lines. As shown in Figure 6a, the cell viability of parental (P) HuH7, Hep3B, HepG2, and SK-Hep1 cells decreased in a dose-dependent manner following sorafenib treatment. However, sorafenib-resistant (SR) cells were less sensitive to sorafenib (Figure 6a). Notably, NPC2 and free cholesterol levels were elevated in culture supernatant of sorafenib-resistant cells compared to that of parental cells (Figure 6b,c). This finding suggests that long-term sorafenib treatment-induced acquisition of sorafenib resistance may be more responsible for NPC2 and free cholesterol secretion to the medium.

### 2.6. Serum NPC2 and Free Cholesterol Levels Increase in Xenografts with Acquired Sorafenib Resistance

**Figure 7:**
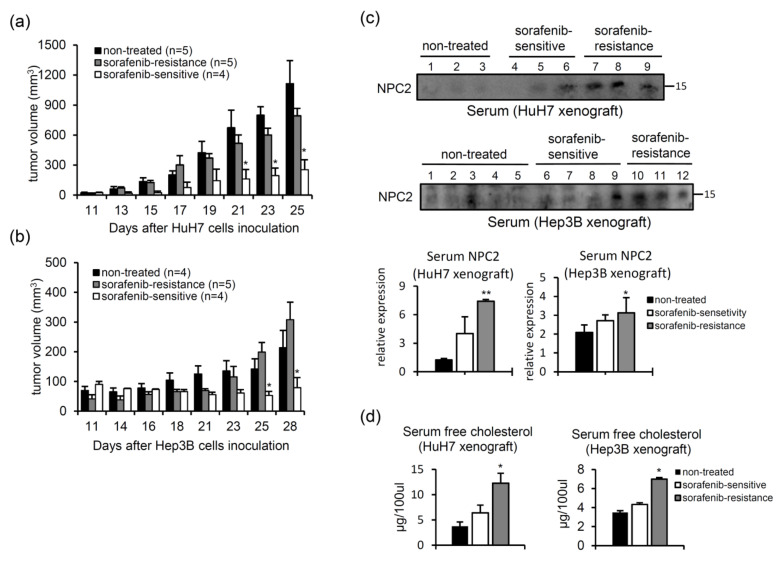
Serum levels of Niemann-Pick type C2 (NPC2) and free cholesterol increase in xenografts with acquired sorafenib resistance. (**a**,**b**) HuH7 and Hep3B cells were subcutaneously injected into NOD/SCID mice, and sorafenib was intraperitoneally injected every day. The mean tumor volume ± SEM at the indicated time is shown. The growth of sorafenib-treated tumors can be divided into two patterns: sorafenib sensitive (white bar) and a lack of response to sorafenib (gray bar, sorafenib resistance). (**c**) Western blot images and quantification of serum NPC2 are shown. Each line was loaded with 100 µg of protein. (**d**) Serum levels of free cholesterol. * *p* < 0.05, ** *p* < 0.01 compared to the black bar.

Since secreted NPC2 and free cholesterol levels were associated with sorafenib resistance in vitro, we next investigated relationships of circulating NPC2-mediated free cholesterol levels between sorafenib-sensitive and sorafenib-resistant xenografts, to define NPC2’s role as a possible circulating biomarker. To mimic the development of acquired sorafenib resistance in advanced HCC patients, we subcutaneously inoculated NOD/SCID mice with HuH7 or Hep3B cells and then treated them with sorafenib. As shown in Figure 7a,b, the tumor growth rate was fastest in the untreated control group. In the sorafenib-treated groups, some tumors grew slowly; thus, we defined them as sorafenib-sensitive. Others grew quickly, which we, thus, defined as sorafenib-resistant (Figure 7a,b). Next, we compared serum NPC2 and free cholesterol levels in these mice by western blotting. As shown in Figure 7c, higher serum NPC2 levels were observed in the sorafenib-resistant groups. In addition, sorafenib-resistant tumor-bearing mice also had higher serum free cholesterol levels (Figure 7d).

## 3. Discussion

Recently, much research has emphasized the effects of cholesterol accumulation on cancer development [31]. Cholesterol accumulation is associated with increased cancer cell survival, decreased cancer cell apoptosis ability [32,33], and enhanced tumor formation [34]. In this study, we showed that free cholesterol accumulation attenuated sorafenib’s efficacy in HCC cells (Figure 2). Since higher cholesterol levels are associated with cancer progression, it was anticipated that lowering excess cholesterol in cancer cells may act as an advantageous anticancer strategy. Several cholesterol-depleting agents showed anticancer effects [35]. Methyl-β-cyclodextrin, a cholesterol-depleting agent, increased the efficacy of tamoxifen chemotherapy in melanomas [36]. In our study, we showed that HPBCD, a free cholesterol-lowering agent used to treat NPC disease, improved the efficacy of sorafenib in a high-cholesterol diet-fed xenograft model (Figure 4d–f). Currently, sorafenib is still the first-line drug for advanced HCC patients. It is important to note that sorafenib needs to bind to lipid membranes and is inserted into the lipid-water interface of the bilayer. This embedding into the membrane disturbs the bilayer structure, leading to increased permeability of the membrane by polar molecules. The extent of this effect depends on the membrane’s lipid composition, such as phosphatidylcholine and cholesterol [37]. In addition, recent publications showed that sorafenib treatment not only inhibited Ras-Raf/VEGF signaling, but also influenced other metabolic pathways, including mitochondrial respiration and cholesterol metabolism. Sorafenib treatment activated AMPK and acted as a mitochondrial uncoupler, which suppressed NASH progression [38]. The Chinese medicinal herb, emodin, sensitizes HCC cells to sorafenib treatment through suppressing cholesterol metabolism [16]. Furthermore, upregulating stearoyl-CoA desaturase (SCD1), a lipogenesis pathway-related enzyme, was associated with the development of sorafenib resistance [39]. These data imply that long-term sorafenib exposure may alter lipogenesis-related gene expressions; on the other hand, the cholesterol status may also affect sorafenib’s effectiveness.

Although α-fetoprotein (AFP) was reported to be an independent surrogate end point for survival, which was evaluated together with the radiological response in sorafenib-treated HCC patients, serum AFP levels are not a pretreatment characteristic which can be used to predict responsiveness to sorafenib [40]. Serum lipid profile analyses found that phosphatidylcholine, cholesterol ester, acylcarnitine, linoleic acid, and diacylglycerol were associated with the response rate in sorafenib-treated patients [41,42]; however, lipid metabolism-related proteins are rarely found in serum. The liver is the main organ regulating cholesterol catabolism into bile [43]. Hepatic NPC2 is a free cholesterol-binding protein that can be secreted into the plasma and bile [28]. In the present study, we found that sorafenib treatment resulted in NPC2 and free cholesterol secretion by HCC cells (Figure 5), suggesting that sorafenib administration may alter lipid catabolism in liver cells. Indeed, some lipid metabolism-related proteins, such as SCD1 [39], sphingomyelin synthase [44], and peroxisome proliferator-activated receptor-δ [45], were reported to predict the response to sorafenib in HCC. In this study, we further demonstrated that the secretion of NPC2 and free cholesterol increased in long-term sorafenib treatment-induced acquired drug resistance of cells and sera (Figure 6 and Figure 7). Previous studies indicated that free cholesterol can mobilize from lysosomes and the expression signal of vesicular lysosomal NPC2 diminishes; however, a certain amount of NPC2 is still present in cells [24,46]. Accordingly, NPC2 may travel with free cholesterol through the cell and perhaps even be secreted with it. Extracellular vesicles play important roles in intracellular and even intercellular communication [47]. Exosomes are a subtype of extracellular vesicles that are released by fusion of multivesicular bodies with plasma membrane [48]. Therefore, lysosome dysfunction may trigger NPC2 or free cholesterol release by extracellular vesicles from liver cells under the sorafenib-resistance condition. Extracellular vesicles have been found to allow the transport of two major developmental signaling pathways: Hedgehog and Wnt. These signaling undergo crucial post-translational lipid modifications, which anchor them to membranes and impede their free release into the extracellular space [49]. Aberrant Hedgehog signaling has been associated with tumorigenesis in many cancers [50,51], as well as NPC disease [52,53,54]. Further research is required to examine the roles of Hedgehog signaling in the secretion of NPC2 and free cholesterol in sorafenib resistant cells. Since liver cells are the main source of plasma and biliary NPC2, the increased serum NPC2 in sorafenib-resistant patients may be related to severe damage to hepatocytes when drug resistance occurs. Our previous study also showed that changes in the glycosylated pattern of NPC2 in serum were associated with cirrhosis and liver cancer [55]. These data imply that secretory NPC2 and free cholesterol may potentially be useful for personalized precision medicine in diagnosing the sorafenib response and developing anti-sorafenib-resistant liver cancer pharmaceuticals.

## 4. Materials and Methods

### 4.1. Cell Culture and Treatments

The human Hep3B, HepG2, HuH7, and SK-Hep1 HCC cell lines were cultured in Dulbecco’s modified Eagle’s medium (DMEM; Gibco, Grand Island, NY, USA) supplemented with 10% fetal bovine serum (FBS) (HyClone, Logan, UT, USA), streptomycin (100 μg/mL), penicillin (100 U/mL), nonessential amino acids (0.1 mM), and L-glutamine (2 mM) at 37 °C in a 5% CO_2_ incubator. Both HuH7 and Hep3B belong to well-differentiated HCC lines, while HepG2 belongs to the hepatoblastoma line. In addition, HuH7 and Hep3B are positive for the presence of HCV replicon and HBV viral DNA, respectively. In contrast, there is no evidence of a hepatitis viral genome in HepG2 cell. Since HuH7 cells express the lowest level of NPC2 and SK-Hep1 express the highest level of NPC2, HuH7 cells was selected to over-express NPC2 and SK-Hep1 was selected to knock down NPC2. The establishment of cell lines with stable NPC2 overexpression and knockdown was previously described [23]. 

HCC cells were seeded in six-well plates and treated with indicated concentrations of U18666A (Sigma-Aldrich, St. Louis, MO, USA) and sorafenib (ApexBio, Houston, TX, USA) for 48 h. To study the secretion of NPC2, cells were treated with a bRaf inhibitor (PLX-4720, 1 μM) or AKT inhibitor (GDC-0941, 1 μM). After 24 h, cells and culture medium were collected for western blot analyses.

### 4.2. Generation of Sorafenib-Resistant (SR) Cells

Hep3B, HepG2, Huh-7, and SK-Hep-1 cells were exposed to a low concentration (2.5 μM) of sorafenib. We enhanced the dose of sorafenib until the cells grew stably. Finally, SR cells (Hep3B-SR, HepG2-SR, HuH7-SR, and SK-Hep1-SR) were cultured in medium containing 7–9 μM sorafenib.

### 4.3. Western Blot Experiments

Cellular proteins (20 μg) or culture medium (50 μL) were loaded in each well, and then separated by sodium dodecylsulfate polyacrylamide gel electrophoresis (SDS-PAGE) and transferred onto polyvinylidene difluoride (PVDF) membranes. The following antibodies used in this study were purchased from Cell Signaling (Beverly, MA, USA): phosphorylated (p)- and total (T)-AKT, ERK, c-Jun N-terminal kinase (JNK), p38, and MAPK kinase (MEK). NPC2 was purchased from Santa Cruz Biotechnology (Santa Cruz, CA, USA). The dilution of primary antibodies is 1:1000. Bands were visualized by an enhanced chemiluminescence (ECL) detection reagent (Millipore, Billerica, MA, USA), and immunoblot signals were quantified by densitometric scanning (ImageJ software 1.47v, National Institutes of Health, Bethesda, MD, USA).

### 4.4. Cell Viability Assay

Cell viability was monitored with a 3-(4,5-dimethylthiazol-2-yl)-2,5-diphenyl tetrazolium bromide (MTT) assay. Cells were seeded in a 96-well plate at a density of 3 × 10^3^ cells/well. After incubation with various concentrations of sorafenib for 48 h, 50 μL of the MTT solution (5 mg/mL) was added to the medium for further incubation for 3 h. Then, 100 μL DMSO was added to each well. Absorbance of the colored solution was measured at an optical density (OD) of 570 nm with a microplate reader.

### 4.5. Free Cholesterol Quantification

The free cholesterol level was measured with a Cholesterol Assay Kit (BioVision, Milpitas, CA, USA) according to the procedure. Cells were treated with indicated doses of sorafenib for 48 h, and lipids were extracted with 200 μL of a chloroform, isopropanol, and NP-40 (7:11:0.1) mixture. After 10 min of centrifugation at 15,000 rpm, the organic phase was dried at 50 °C for 10 min to remove chloroform and under a vacuum for 30 min to remove the organic solvent. Dried lipids were dissolved with 200 μL of Cholesterol Assay Buffer by sonication until the solution became cloudy. Mixed reagents were incubated for 60 min at 37 °C in the absence of light, and the absorbance was measured at 570 nm with an enzyme-linked immunosorbent assay (ELISA) reader.

### 4.6. Animal Experiments

(A) In the first HCC xenograft model, we compared the tumor growth rates between animals fed a normal diet and those fed a 2% high-cholesterol diet (Table 1). Six–seven-week-old female NOD/SCID mice were purchased from the National Laboratory Animal Center (Taipei, Taiwan). Mice were randomly divided into two groups: a normal diet and a high-cholesterol diet for pre-administration for 12 days, after which HuH7 cells (10^6^) were subcutaneously inoculated into each mouse (Figure 4a). The tumor volume was measured three times per week using Vernier calipers.
(B) Next, we studied the effects of a high-cholesterol diet plus a free cholesterol-lowering drug in a sorafenib-treated HCC xenograft model (Figure 4d). After 12 days of feeding mice a high-cholesterol diet, HuH7 cells (10^6^) were subcutaneously injected into NOD/SCID mice. Then, the mice were divided into two groups: (1) sorafenib treatment (intraperitoneal injection 25 mg/kg, six times per week) and (2) sorafenib (intraperitoneal injection 25 mg/kg, six days per week) combined with 2-hydroxypropyl-β-cyclodextrin (HPBCD) treatment (intraperitoneal injection 4000 mg/kg, twice weekly, Sigma-Aldrich). HPBCD is currently in phase 2 clinical trials for evaluation of its effectiveness in treating NPC2 deficit-associated free cholesterol accumulation in Niemann-Pick type C disease [27,56,57]. The tumor volume was measured three times per week using Vernier calipers. Water intake and food intake were measured twice a week.
(C) Xenograft HCC model of acquired sorafenib resistance. HuH7 and Hep3B cells (10^6^) were subcutaneously injected into NOD/SCID mice, and sorafenib (25 mg/kg) was intraperitoneally injected every day. The tumor volume was measured three times per week using Vernier calipers. After 25 (HuH7) or 28 days (Hep3B), animals were sacrificed to collect tumor tissues and serum. Serum samples (100 μg) were subjected to a western blot analysis.

All the individual tumor volumes (TV) were calculated using the formula: TV = (L × W^2^)/2, wherein length (L) is the longest diameter and width (W) is the shortest diameter perpendicular to the length. The researcher processed all the steps under the same conditions.

### 4.7. Statistical Analysis

Data are expressed as the mean ± standard deviation (SD) or standard error of the mean (SEM). Statistical analyses were performed by a two-way ANOVA and Bonferroni post hoc analyses to examine significant differences using SPSS v20.0 software (SPSS Inc, Chicago, IL, USA). A statistically significant difference was considered at *p* < 0.05.

## 5. Conclusions

HCC is a major cause of cancer-related death worldwide. For late-stage disease, sorafenib is the only first-line drug approved by the FDA for systemic therapy. Despite the capacity of sorafenib to increase the survival of HCC patients, the development of resistance to this drug has raised concerns in recent years. Therefore, identifying the secretable biomarkers that can predict sorafenib response will provide a more precise treatment strategy for advanced HCC patients. NPC2, a secreted protein, plays an important role in regulating intracellular free cholesterol homeostasis. In this study, we showed that NPC2 downregulation-mediated free cholesterol accumulation attenuated sorafenib-induced cytotoxicity through enhancing MAPK and AKT signaling in HCC cells. In vivo, a high-cholesterol diet enhanced the tumor growth rate, while HPBCD treatment, which reduced free cholesterol accumulation, improved sorafenib’s tumor-inhibiting ability. To further evaluate whether NPC2-mediated free cholesterol levels can act as a predictive factor for sorafenib susceptibility, we showed that sorafenib treatment increased NPC2 and free cholesterol secretion through inhibiting Raf/MEK/ERK signaling. Furthermore, NPC2 and free cholesterol levels increased in sorafenib-resistant cultured supernatant and in sera of acquired sorafenib-resistant xenografts. Our cell-based and animal-based models may provide more information on the diagnosis/prediction of the sorafenib response in each advanced HCC patient and achieve the personalized precision medicine goal in the future.

## Figures and Tables

**Table 1:** Composition of the normal and high-cholesterol diets.

Ingredient	Normal Diet g/kg	High Cholesterol Diet g/kg
Cornstarch	465	440
Maltodextrin	155	155
Sucrose	100	100
Casein	140	140
L-Cysteine	2	2
Fresh soybean oil	40	40
Cellulose	50	50
Mineral mix (AIN-93M-MI)	35	35
Vitamin mix (AIN-93-VX)	10	10
Choline bitartrate	3	3
Cholesterol		20
Sodium cholate		5
Total	1000	1000

## Data Availability

Resources and reagents can be requested from the corresponding author.
